# Comment on Sklifasovskaya et al. Hypertension and Diabetes Cooperatively Drive HSP90 Activation, HSP70 Suppression, and Left Ventricular Interstitial Expansion: Relevance to Maladaptive Myocardial Remodeling. *Pathophysiology* 2026, *33*, 19

**DOI:** 10.3390/pathophysiology33030046

**Published:** 2026-07-06

**Authors:** Bişar Amaç

**Affiliations:** Department of Perfusion, Faculty of Health Sciences, Harran University, Sanliurfa 63300, Turkey; amacbisar@gmail.com

We read with great interest the article by Sklifasovskaya et al., entitled “Hypertension and Diabetes Cooperatively Drive HSP90 Activation, HSP70 Suppression, and Left Ventricular Interstitial Expansion: Relevance to Maladaptive Myocardial Remodeling” [1]. The authors provide valuable experimental data on the differential regulation of HSP90 and HSP70 in the left ventricular myocardium under arterial hypertension, streptozotocin-induced diabetes, and their comorbidity. The study is particularly noteworthy for emphasizing the potential role of heat shock protein imbalance in cardiometabolic myocardial stress and structural remodeling.

However, we believe that several methodological issues should be considered when interpreting the findings.

First, although the study provides detailed histological and molecular data, in vivo cardiac function was not assessed. The authors also acknowledge this limitation, stating that their ethical protocol was restricted to terminal histological and molecular endpoints and did not include in vivo cardiac function or systemic biomarkers. Therefore, while the observed HSP90 upregulation, HSP70 suppression, and interstitial expansion may be associated with myocardial stress and structural remodeling, their direct relationship with systolic or diastolic dysfunction remains unproven. Accordingly, the findings should be interpreted primarily as molecular and histological correlations rather than functional evidence of maladaptive myocardial remodeling.

Second, the severity of the diabetes model may have influenced the molecular response observed in the study. The authors reported that, after confirmation of diabetes, non-fasted blood glucose levels in diabetic animals ranged between 25.5 and 33.3 mmol/L during the 30-day experimental period, and no insulin therapy was administered. In addition, ketonuria and glucosuria were evaluated in streptozotocin-induced diabetic animals. These findings suggest that the model may represent not only diabetes but also severe hyperglycemic and catabolic metabolic stress. Therefore, it would be useful to clarify whether the pronounced HSP90 activation and HSP70 suppression in the AH+DM group reflect a specific synergistic interaction between hypertension and diabetes or, at least in part, the effect of uncontrolled severe metabolic stress.

Third, some technical details in the RT-qPCR methodology require clarification. The methods section states that HSP70 and HSP90 mRNA expression was quantified using a SYBR-based qPCR mix; however, the same paragraph also refers to TaqMan technology. Moreover, although the study focuses on HSP70 and HSP90, the relative abundance of “HSP60 gene” mRNA is mentioned in the RT-qPCR description. These inconsistencies may create confusion regarding the molecular methodology and should be clarified to improve reproducibility and methodological transparency.

Fourth, the statistical approach to multiple comparisons appears unclear. The authors state that *p*-values were adjusted using the Holm–Bonferroni method and that all reported significant differences remained significant after correction. However, the same section also states that, given the hypothesis-driven design and small sample size, post hoc correction for multiple comparisons was not applied. This creates ambiguity regarding the exact correction strategy used. In a preclinical study with only five animals per group, a clear and internally consistent explanation of the multiple-comparison procedure is important for evaluating the robustness of the reported differences.

For these reasons, our observations aim to highlight points that may require further clarification by the authors, particularly regarding functional cardiac assessment, the metabolic severity of the diabetes model, RT-qPCR reporting, and the multiple-comparison strategy. We believe that clarifying these issues will help readers to interpret the HSP70/HSP90 response and its relationship with dysregulated myocardial remodeling more accurately.
